# People with multiple unhealthy lifestyles are less likely to consult primary healthcare

**DOI:** 10.1186/1471-2296-15-126

**Published:** 2014-06-26

**Authors:** Xiaoqi Feng, Federico Girosi, Ian S McRae

**Affiliations:** 1 Centre for Health Research, School of Medicine, University of Western Sydney, Locked Bag 1797, Penrith, NSW 2751, Australia; 2 Australian Primary Healthcare Research Institute, Australian National University, Building 63, Cnr Mills and Eggleston Rds, Canberra, ACT 0200, Australia

## Abstract

**Background:**

Behavioural interventions are often implemented within primary healthcare settings to prevent type 2 diabetes and other lifestyle-related diseases. Although smoking, alcohol consumption, physical inactivity and poor diet are associated with poorer health that may lead a person to consult a general practitioner (GP), previous work has shown that unhealthy lifestyles cluster among low socioeconomic groups who are less likely to seek primary healthcare. Therefore, it is uncertain whether behavioural interventions in primary healthcare are reaching those in most need. This study investigated patterns of GP consultations in relation to the clustering of unhealthy lifestyles among a large sample of adults aged 45 years and older in New South Wales, Australia.

**Methods:**

A total of 267,153 adults participated in the 45 and Up Study between 2006 and 2009, comprising 10% of the equivalent demographic in the state of New South Wales, Australia (response rate: 18%). All consultations with GPs within 6 months prior and post survey completion were identified (with many respondents attending multiple GPs) via linkage to Medicare Australia data. An index of unhealthy lifestyles was constructed from self-report data on adherence to published guidelines on smoking, alcohol consumption, diet and physical activity. Logistic and zero-truncated negative binomial regression models were used to analyse: (i) whether or not a person had at least one GP consultation within the study period; (ii) the count of GP consultations attended by each participant who visited a GP at least once. Analyses were adjusted for measures of health status, socioeconomic circumstances and other confounders.

**Results:**

After adjustment, participants scoring 7 unhealthy lifestyles were 24% more likely than persons scoring 0 unhealthy lifestyles not to have attended any GP consultation in the 12-month time period. Among those who attended at least one consultation, those with 7 unhealthy lifestyles reported 7% fewer consultations than persons with 0 unhealthy lifestyles. No effect modification was observed.

**Conclusion:**

To optimise the prevention of lifestyle-related diseases, interventions for positive behavioural change need to incorporate non-primary healthcare settings in order to reach people with multiple unhealthy lifestyles.

## Background

To prevent type 2 diabetes mellitus (T2DM) and other lifestyle-related diseases, governments have issued guidelines on physical activity, diet, alcohol and smoking [1-4] and funded interventions focussed upon positive lifestyle change [5]. These interventions are often implemented by general practitioners (GPs) within primary healthcare settings, but this strategy could be problematic if the people most in need, in particular people with multiple unhealthy lifestyles who have not yet developed chronic diseases, do not attend these settings. Evidence shows that people engaged in a higher number of unhealthy lifestyles are more likely to have poor health literacy [6], experience low socioeconomic circumstances (e.g. low incomes and few educational qualifications) and live in deprived and remote locations [7-11]. Although these factors suggest that people with multiple unhealthy lifestyles and no diagnosed chronic condition will be less likely to consult a GP and to receive the intervention that was designed to help them, to what extent this has been the case remains unclear since these lifestyles are also associated with chronic conditions that necessitate regular consultations, such as T2DM.

Previous work has shown that people who smoke tobacco consult a GP less frequently (e.g. [12]), but there has not been any similar research so far which has looked at multiple unhealthy lifestyles and GP consultations. Earlier work on consultation duration has been conducted in relation to socioeconomic circumstances, with persons from less favourable socioeconomic backgrounds often having shorter time with a GP [13,14]. Although longer consultation duration may serve as a proxy for patient-GP interactions that involve preventive health advice, the more basic question of whether attendance for GP consultations of any duration is associated with multiple unhealthy lifestyles remains unknown. Accordingly, the purpose of this paper is to investigate whether or not people with multiple unhealthy lifestyles are less likely to consult a GP, and to what degree such an association is related to health status and measures of socioeconomic and geographical circumstances.

## Methods

### Data

This study used data on the 267,153 respondents to the 45 and Up Study, a survey carried out between 2006 and 2009 on the health and social wellbeing of individuals aged 45 years and older living in New South Wales (NSW), Australia [15]. Participants were randomly sampled from the Medicare Australia enrolment database held by the Department for Human Services (formerly ‘Medicare’), the national provider of universal health insurance in Australia. Eligible individuals were mailed the questionnaire, an information sheet and a consent form and provided with a reply paid envelope. The survey over-sampled individuals aged 80 years and over and residents of rural areas by a factor of two. In addition, all residents aged 45 years and older in remote areas were sampled. People could also voluntarily join the study by requesting an information pack via a telephone helpline, although this group constitutes a very small fraction of the participants. The 45 and Up Study had an overall response rate of 18%, comprising approximately 10% of all persons of 45 years or older living in NSW. Although the response rate is low and participants tended to be of more favourable socioeconomic circumstances than average for the age group, previous work has shown that analytical findings based on internal comparisons, such as odd-ratios, are generalizable and comparable to those derived from smaller but more ‘representative’ population health surveillance [16].

Participants gave permission for their survey responses to be linked to a variety of data, including the Medicare Benefits Schedule (MBS). Linkage between the 45 and Up Study and MBS data for the period 2003–2012 was performed by the Sax Institute under approvals from the Medicare Australia and Australian Government Department of Health ethics committee. The linkage was direct (i.e. ‘deterministic’) using an encrypted unique identification number for each participant provided by the Department of Human Services and based on the Medicare number. The linked data were accessed and analysed through a secure facility which is managed by the Sax Institute. The 45 and Up Study was approved by the Department of Health and Ageing Departmental Ethics Committee and by the University of New South Wales Human Research Ethics Committee (HREC). Ethical approval for this particular study was provided by the NSW Population and Health Services Research Ethics Committee and the University of Western Sydney HREC.

### GP attendance variables and statistical analysis

The main variables of interest in this study were derived from GP consultations available for every participant via linked MBS claims data. GP attendance was defined by the following MBS claims for: (i) ‘A1-GP attendances (3, 23, 36, 44); (ii) ‘Medical Practitioner other than GP’ (52, 53, 54, 57). Since survey participants were interviewed at different points in time between 2006 and 2009 we defined the window of observation for GP consultations by extracting MBS claims data for the period 6 months prior and 6 months post survey completion for each participant. The choice of a one-year window of observation was motivated by the need to strike a balance between sample size and validity of the observations; the longer the time period, the larger the number of GP attendances, but also the greater the likelihood that participants’ circumstances have changed and the health survey no longer adequately reflects their health and social circumstances.

Two main variables were measured from this MBS claims data. The first variable, which was defined for the whole sample, was a binary response reflecting whether a participant consulted any GP at least once within a one year period. Participants consulting at least one GP were assigned ‘0’ and those who did not report any consultation were assigned a ‘1’. This binary variable was modelled using logistic regression and associations with other variables were assessed using odds ratios (OR) with 95% confidence intervals (95% CI).

The second outcome, which was defined only for the participants with at least one GP attendance over the year, was the yearly count of GP attendances. This variable was modelled using zero-truncated negative binomial regression, since its minimum value is one and it is over-dispersed. Associations with other variables were measured using rate ratios (RR) and 95% confidence intervals (95% CI). All regression models were estimated using Stata v.12 (StataCorp, College Station, TX).

### Multiple unhealthy lifestyles

The main explanatory variable was a categorical index of unhealthy lifestyles ranging from 0 to 8, with higher scores indicating more unhealthy lifestyles. The derivation of this index followed previous work with the same dataset [11], summing binary variables denoting non-adherence to published lifestyle guidelines [1-4]. The components of the index and their definitions are shown in Table 1.

**Table 1:** Components of the unhealthy lifestyle index

**Index Component**	**Lifestyle activity measured**
Smoking	Smoking within the past year
Alcohol	Consuming two or more alcoholic drinks a day
Moderate-to-vigorous physical activity (MVPA)	Not participating in at least 30 minutes of MVPA on 5 or more days a week
Fruit	Consuming less than two fruit serves per day
Vegetables	Consuming less than five vegetables portions per day
Meat	Consuming more than 5 or less than 3 weekly portions of red meat, or consuming more than one weekly portion of processed meat.
Milk	Consuming milk which is not reduced fat or skim, or not consuming any milk
Fish	Consuming less than three weekly portions of fish

An individual-level binary variable was created for each of the rows in this table. The variable took the value of 1 if the activity described in the right column was observed.

### Adjustment for confounding variables and other sources of bias

The analysis controlled for many confounders, including health and socioeconomic status. Health status was measured using self-rated health, mental health, weight status, and the number of medically-diagnosed chronic health problems. Self-rated health was assessed using the question ‘In general, how would you rate your health overall?’ in which the participants were asked to circle an appropriate response from 5 options (aggregated into 0 = excellent, very good, good, or fair; 1 = poor). Mental health was proxied using the Kessler 10 psychological distress scale, with scores of 22 and over indicative of participants experiencing psychological distress [17]. Weight status was derived from self-reported height and weight to construct body mass index, from which participants were classified as normal weight, overweight, obese, or underweight in line with World Health Organisation recommendations [18]. Participants were asked to self-report whether a doctor had ever told them that they had a range of chronic health problems, including cardiovascular diseases, diabetes, cancers, high blood pressure, Parkinson’s disease and stroke. Rather than considering each of these chronic conditions separately, we summarize them with a variable that simply counts how many of them are present.

Socioeconomic and geographical circumstances were measured using indicators of household income, employment status, educational qualifications, neighbourhood remoteness and deprivation [19]. Other possible confounders included were age, gender, couple status and country of birth. These variables were used as covariates in the multivariate regression models for our two primary outcomes. Therefore for each outcome we have run two sets of regression models: in the unadjusted models we regress the outcome against the unhealthy lifestyle index only, while in the adjusted models we also include as covariates the confounders listed above. Standard errors were adjusted for the clustering of participants within neighbourhoods using the Huber White method [20].

## Results

In Table 2 we report unadjusted summary statistics on the two outcome variables (columns 3 and 4) and the unhealthy lifestyle index (column 1). The table shows that the largest groups of participants displayed between 2 and 5 unhealthy lifestyles. Those with more unhealthy lifestyles were more likely to spend 12 months without consulting any GP. Among those who did seek primary healthcare, participants with more unhealthy lifestyles consulted GPs on fewer occasions. The pattern of GP consultations by each individual component of the unhealthy lifestyle index, as reported in Table 3 showed a significant difference between those who did and did not meet guidelines in all but one case of 16 comparisons. The differences were markedly greater in relation to seeing or not seeing a GP than the mean number of consultations, with differences ranging from 5% more seeing a GP to 28% more seeing a GP, which are material differences. For each individual lifestyle component tested, other than the MVPA, those who do not adhere to guidelines were more likely not to have seen a GP during the 12 month window of observation. For example, 8.8% of tobacco smokers did not see at least one GP within the 12 month study period, compared to 7.8% of non-smokers. Unlike findings from the overall unhealthy lifestyle index, the mean count of GP consultations among participants who saw at least one GP in the study period was not consistently lower among those who did not meet individual published guidelines. Participants who exceeded alcohol consumption guidelines reported fewer GP consultations than those keeping to the guideline, for example, whereas, those who achieved at least 150 minutes of MVPA a week reported fewer GP consultations in comparison to their more sedentary counterparts.

**Table 2:** General practitioner consultations attended and the unhealthy lifestyle index

**Number of unhealthy lifestyles**	**Sample: N (%)**	**% not seeing at least one general practitioner within 12 months**	**Mean count of consultations among those who attended at least one consultation with a general practitioner within 12 months**
0 (ref)	3,225 (1.5%)	6.5	6.9
1	16,751 (7.7%)	6.5	6.8
2	38,959 (17.9%)	6.6	6.8
3	55,948 (25.7%)	7.5*	6.8
4	51,663 (23.8%)	8.3***	6.7
5	32,760 (15.1%)	9.4***	6.6**
6	13,869 (6.4%)	10.1***	6.4***
7	3,779 (1.7%)	11.0***	6.1***
8	423 (0.2%)	16.4***	5.9***

***p < 0.001; **p < 0.01; *p < 0.05.

For each number of unhealthy lifestyles we compared the means of the outcome variables to the mean in the reference category (individuals with no unhealthy lifestyles) and report the corresponding significance in terms of p-values.

**Table 3:** General practitioner consultations attended and components of the unhealthy lifestyles index

**Unhealthy lifestyle index component**	**% not seeing at least one general practitioner within 12 months**	**Mean count of consultations among those who attended at least one consultation with a general practitioner within 12 months**
**Tobacco smoking**		
Smoking within the past year (ref)	8.8%	7.1
Did not smoked within the last 12 months	7.8%***	6.9***
**Alcohol**		
> = 2 alcoholic drinks a day (ref)	9.1%	5.9
< 2 alcoholic drinks a day	7.6%***	7.2***
**MVPA**^**†**^		
< 30 minutes of MVPA on > =5 days a week (ref)	7.9%	7.2
> = 30 minutes of MVPA on > =5 days a week	8.2%**	6.4***
**Fruit**		
< 2 fruit serves a day (ref)	8.6%	6.8
> = 2 fruit serves a day	7.4%***	7.0***
**Vegetables**		
< 5 vegetables portions a day (ref)	8.0%	6.9
> = 5 vegetables portions a day	6.6%***	7.1***
Red and processed meat		
<3 or >5 red meat or > 1 processed meat a week (ref)	8.7%	7.0
> = 3 & < =5 red meat or < = 1 processed meat a week	7.7%***	6.9
**Milk**		
Whole or do not drink milk (ref)	9.4%	7.0
Low-fat/skim milk	6.8%***	6.9***
**Fish**		
< 3 portions of fish a week (ref)	8.0%	6.9
> = 3 portions of fish a week	7.6%***	7.2***

^†^MVPA: moderate-to-vigorous physical activity.

***p < 0.001; **p < 0.01; *p < 0.05.

For each component of the index we compared the means of the outcome variables to the mean in the reference category (individuals who do not meet the guidelines) and report the corresponding significance in terms of p-values.

**Figure 1:**
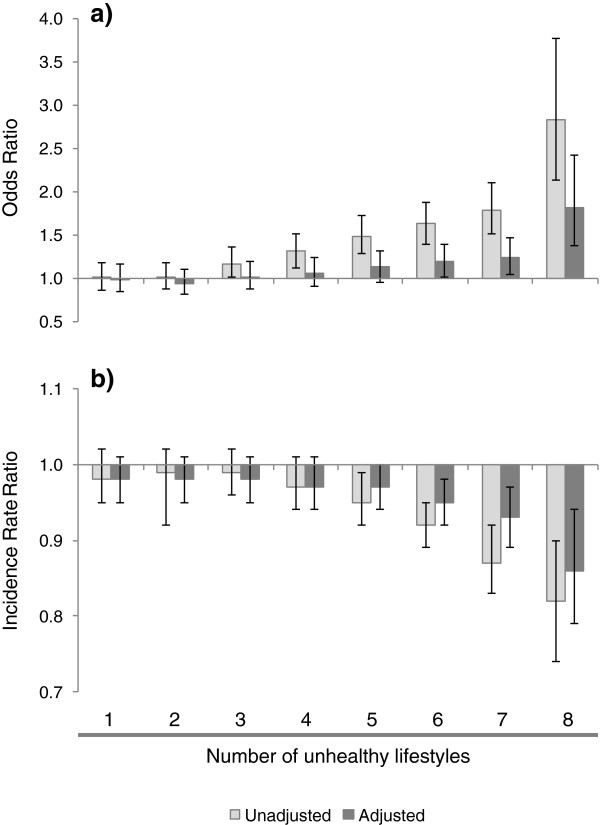
**Association between multiple unhealthy lifestyles and GP consultations.** Reference group: people with zero unhealthy lifestyles and: **a)** the odds of not consulting any GP within 12 months (logistic regression); and **b)** incident rate ratios of the count of consultations attended by those who saw at least one GP within 12 months (zero-truncated negative binomial regression). Bars represent odds-ratios and incidence rate-ratios, while lines represent 95% confidence intervals.

The results in Tables 2 and 3 were based on unadjusted figures. In order to account the confounding factors discussed above, we show in Figure 1 both the unadjusted and adjusted associations between each outcome variable and the index of unhealthy lifestyles. Bars represent odds-ratios (ORs) and incidence rate ratios (RRs) while lines represent 95% confidence intervals (CIs). Before discussing the figure we note that while the index takes values from 0 to 8, there are relatively few people with a value of 8 (423 individuals, as shown in Table 2). Therefore when performing comparisons across index categories it seems more informative to compare the category of people with 7 unhealthy lifestyles, rather than 8, with the reference group, in order to avoid overstating the results and draw conclusions based on the tails of the distribution of unhealthy lifestyles.Figure 1 clearly shows that results are robust to adjustment for health status, socioeconomic circumstances and other potential confounders. Prior to adjustment, for example, participants scoring 7 unhealthy lifestyles had odds of not consulting any GP that were 79% larger than the odds for participants with no unhealthy lifestyles. Similarly, among individuals with at least one GP consultation, those with 7 unhealthy lifestyles reported 13% fewer consultations than persons with no unhealthy lifestyles. Adjustment resulted in substantial attenuation of these effect sizes (down to 24% and 7%, respectively), but the associations remained statistically significant for higher categories on the unhealthy lifestyle index.

Analysis of the regression coefficients shows that in terms of both outcome variables, people in poorer health and in socioeconomically disadvantaged circumstances were more likely to seek GP consultations. For example, the odds of not seeing even one GP within the study period was lower for people experiencing psychological distress (OR 0.89, 95% CI 0.83, 0.95) and with 3 or more chronic diseases (OR 0.49, 95% CI 0.46, 0.53), but higher for those with university-level education (OR 1.44, 95% CI 1.36, 1.53) and household incomes above $70,000 per annum (OR 1.34, 95% CI 1.26, 1.44). Similarly, counts of GP consultations were higher for people experiencing psychological distress (RR 1.24, 95% CI 1.23, 1.26) and with 3 or more chronic diseases (RR 1.65, 95% CI 1.63, 1.67), but lower for those with university-level education (RR 0.84, 95% CI 0.82, 0.85) and household incomes above $70,000 per annum (RR 0.79, 95% CI 0.78, 0.80).

## Discussion

Interventions targeting behavioural change are being implemented within primary healthcare settings. However, this study shows that people with multiple unhealthy lifestyles are among the least likely to consult a GP. This was despite the observation that people with more unhealthy lifestyles reported poorer health and socioeconomic disadvantage, factors which were positively associated with primary healthcare use. These findings extend previous work in NSW using an earlier release of the 45 and Up Study which assessed GP consultation frequency in relation to tobacco smoking [12]. This suggests a need to expand the range of settings in which these interventions are located to reach those persons most in need (e.g. workplaces).

Amid an increasing number of studies exploring the correlates of multiple unhealthy lifestyles, this is the first to investigate associations with GP consultations. Strengths include the large sample size and linkage to records of actual GP consultations. This is crucial, as it eliminates the possibility of bias were participants required to recall the number of GP consultations they had within a 6–12 month period via self-report. Indeed, this linkage afforded the possibility to include prospective GP consultations occurring after the survey, which would not have been possible had self-reported outcomes been used. Descriptive results also demonstrated the potential usefulness of considering an index of unhealthy lifestyles rather than each lifestyle individually, since the mean consultation count was associated with the overall index but not consistently with individual items. There are, however, some limitations that warrant acknowledgement. This is a cross-sectional study, and therefore distinguishing particular causal mechanisms is a challenging process. Although unfavourable socioeconomic circumstances can indicate whether a person will find it difficult to physically access a GP, these measures are also correlated with low levels of health literacy [6]. As such, it was not possible in this study to attribute attenuation in the effect sizes to either deprivation or health literacy, though both are likely to play important roles. Similarly, people with poorer geographic access to primary healthcare may be less likely to consult a GP. No formal measure of geographic access was available, such as travel-time to the nearest GP [21,22], though adjustment for rurality and remoteness of residence will have addressed this issue to some degree.

Although participants with multiple unhealthy lifestyles were less likely to visit primary healthcare, data were unavailable to investigate whether some of those participants sought similar services through hospital admissions, emergency department presentations, allied health practitioners and alternative healthcare providers. This is supported by findings from a recent study in the same geographic area which documented a higher risk of avoidable hospital admissions among people who reported multiple unhealthy lifestyles [23]. For a fuller overview of engagement with the health system, it would be useful for future studies to investigate patterns of overall health service use across primary, secondary and tertiary healthcare [24].

It is important to recognise that although a large proportion of the sample did consult a GP at least once within the 12 months, this does not guarantee that preventive health advice was provided [25,26]. Furthermore, the number of GP consultations is not the only variable that can be used to measure the opportunities for prevention, and the duration of the visits may play a role as well. Longer consultations afford more opportunities for health promotion [27,28], but shorter consultations are more common in low socioeconomic areas where preventive health advice is arguably most in need [13,14]. Therefore, duration is also important, but while the type of visit is easily observed in the Medicare data (e.g. standard versus prolonged), the actual time spent with the patient is not, and estimating it would require some additional assumptions. We focussed exclusively on the number of consultations attended, which is much more accurately measured, while being mindful of the observed pattern of duration. Our work therefore can be extended in the future through investigating the association between consultation duration and unhealthy lifestyles in detail. These are important avenues for optimising evidence-based preventive health policy.

The relationship between unhealthy lifestyles and the number of GP consultations attended may be potentially confounded by a number of other factors. For example it is known, that low SES Australians are more likely to have unhealthy lifestyles and risk factors that are conducive to chronic conditions [11], which in turn contribute to higher primary care utilization. Therefore in this analysis we provided two perspectives: one in which we measure the direct association between unhealthy lifestyle and GP visits, and a second in which we adjusted for a variety of potential confounders, so that the comparison across clusters of unhealthy lifestyles is performed while addressing competing explanations.

## Conclusion

In conclusion, interventions to prevent chronic diseases need to be located across a range of settings to ensure they reach all people who stand to benefit from them.

## Competing interests

The authors declare that they have no competing interests.

## Authors’ contributions

XF contributed to the study design, data analysis, data interpretation and wrote the first draft. FG and IM contributed to the study design, data interpretation and redrafted the manuscript. All authors contributed to editing the final draft. All authors read and approved the final manuscript.

## Pre-publication history

The pre-publication history for this paper can be accessed here:

http://www.biomedcentral.com/1471-2296/15/126/prepub
